# Supplementary material for: The rate and spectrum of mosaic mutations during embryogenesis revealed by RNA sequencing of 49 tissues
Source: Genome Med. 2020 May 27;12:49. doi: 10.1186/s13073-020-00746-1 (PMC7254727; doi:10.1186/s13073-020-00746-1)

**Additional file 2**

**Supplementary tables**

**Table S1**. **STAR alignment parameters.**

**
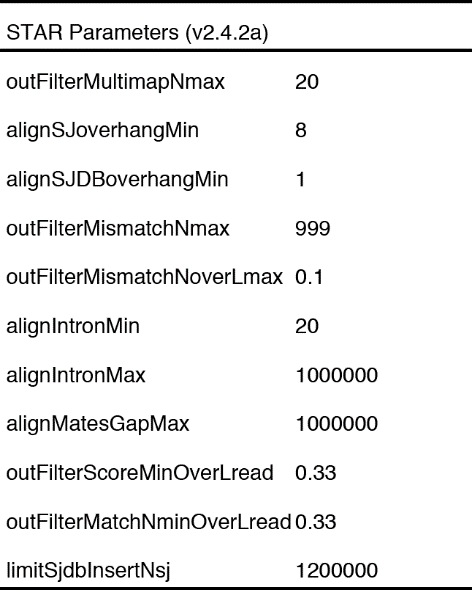
**

**Table S2. Performance of RNA-seq based variant detection in CLL samples using different thresholds for variant allele frequency (VAF).**


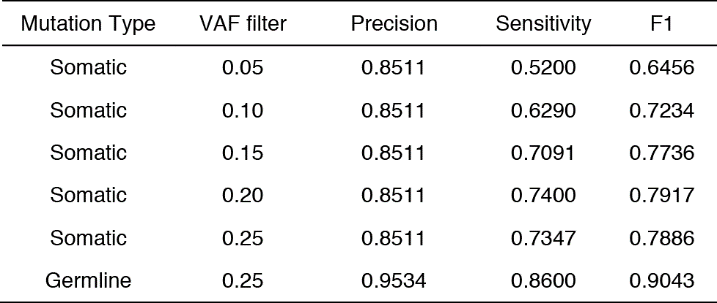


**Table S3.** **Number and rate of EEMMs and MEMMs in the four sets of constitutively expressed genes.**


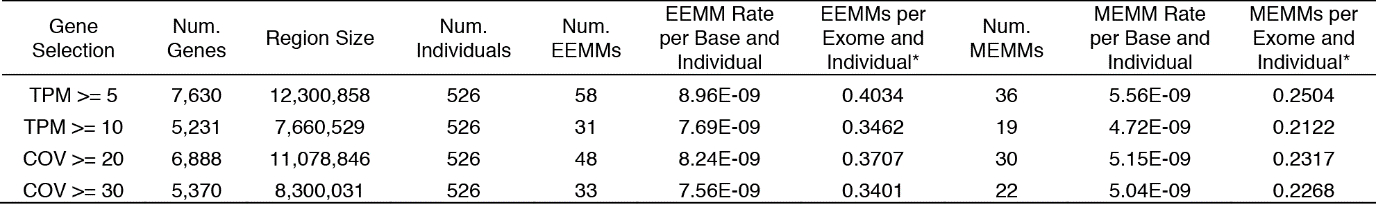


**Table S4**. **Signature of selection in cancer genes. dN/dS values above 1 indicate positive selection.**


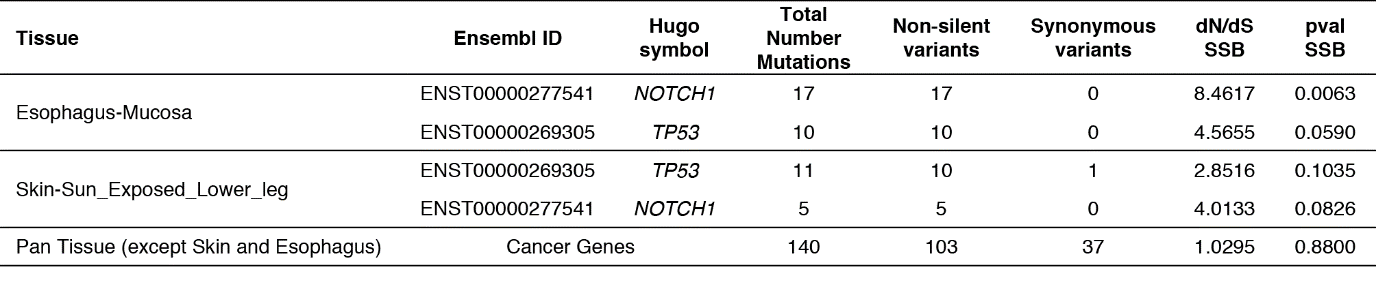

Supplement: Supplementary file 2 — Additional file 2: Supplementary tables. This document contains additional supporting evidences presented as supplemental tables (Table S1-S4). [file 13073_2020_746_MOESM2_ESM.docx]
