# Supplementary material for: The rate and spectrum of mosaic mutations during embryogenesis revealed by RNA sequencing of 49 tissues
Source: Genome Med. 2020 May 27;12:49. doi: 10.1186/s13073-020-00746-1 (PMC7254727; doi:10.1186/s13073-020-00746-1)
Supplement: Supplementary file 4 — Additional file 4: Supplementary figures. This document contains additional supporting evidences presented as supplemental figures (Fig. S1-S15). [file 13073_2020_746_MOESM4_ESM.docx]

**Additional file 4**

**Supplementary figures**

**
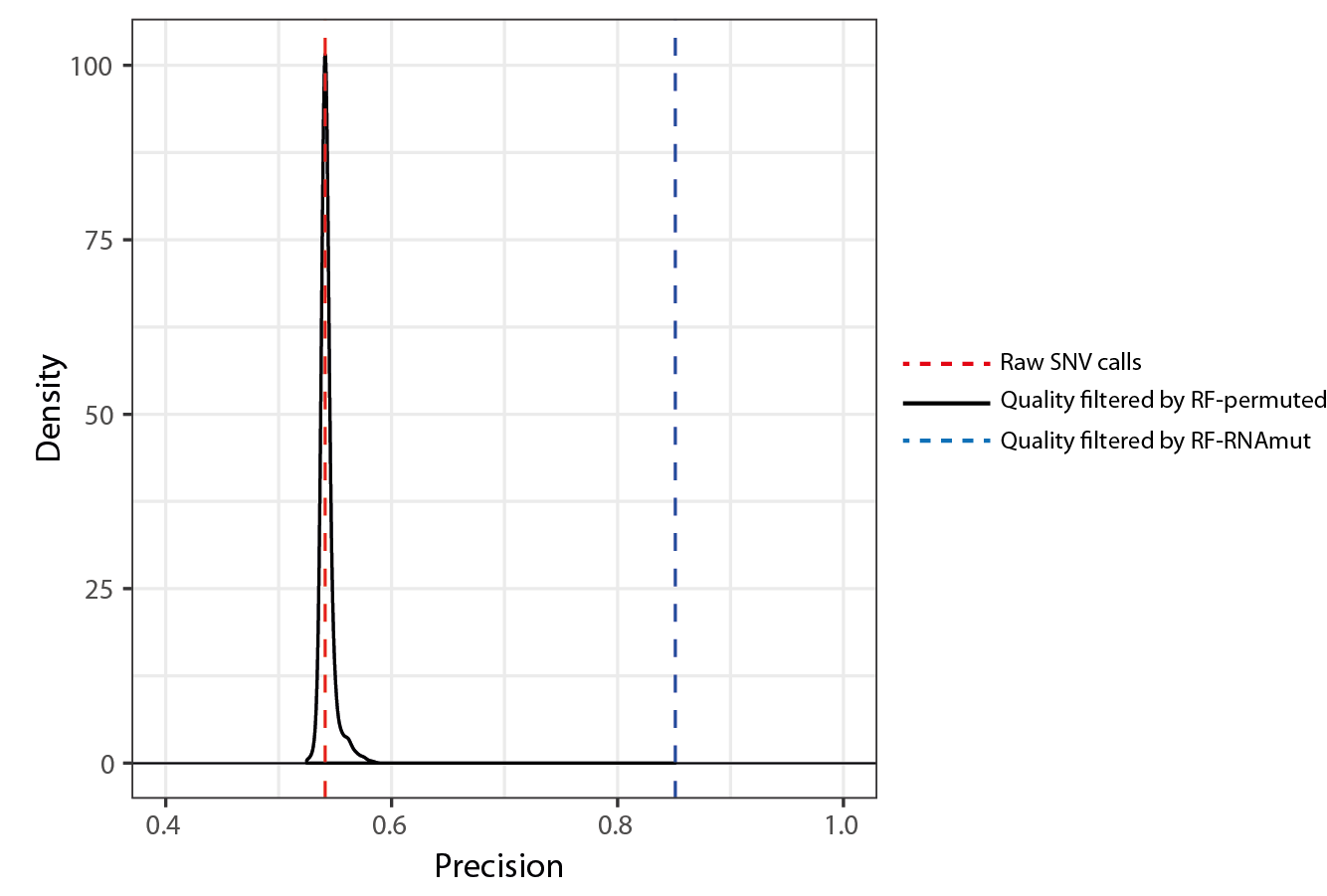
**

**Fig. S1.** Precision of random forest models for variant quality filtration trained on permuted data (500 permutation models, black distribution) compared to the random forest model *RF-RNAmut* applied in our study (blue dashed line). The precision of the raw ‘SNV’ calls is indicated as red dashed line.


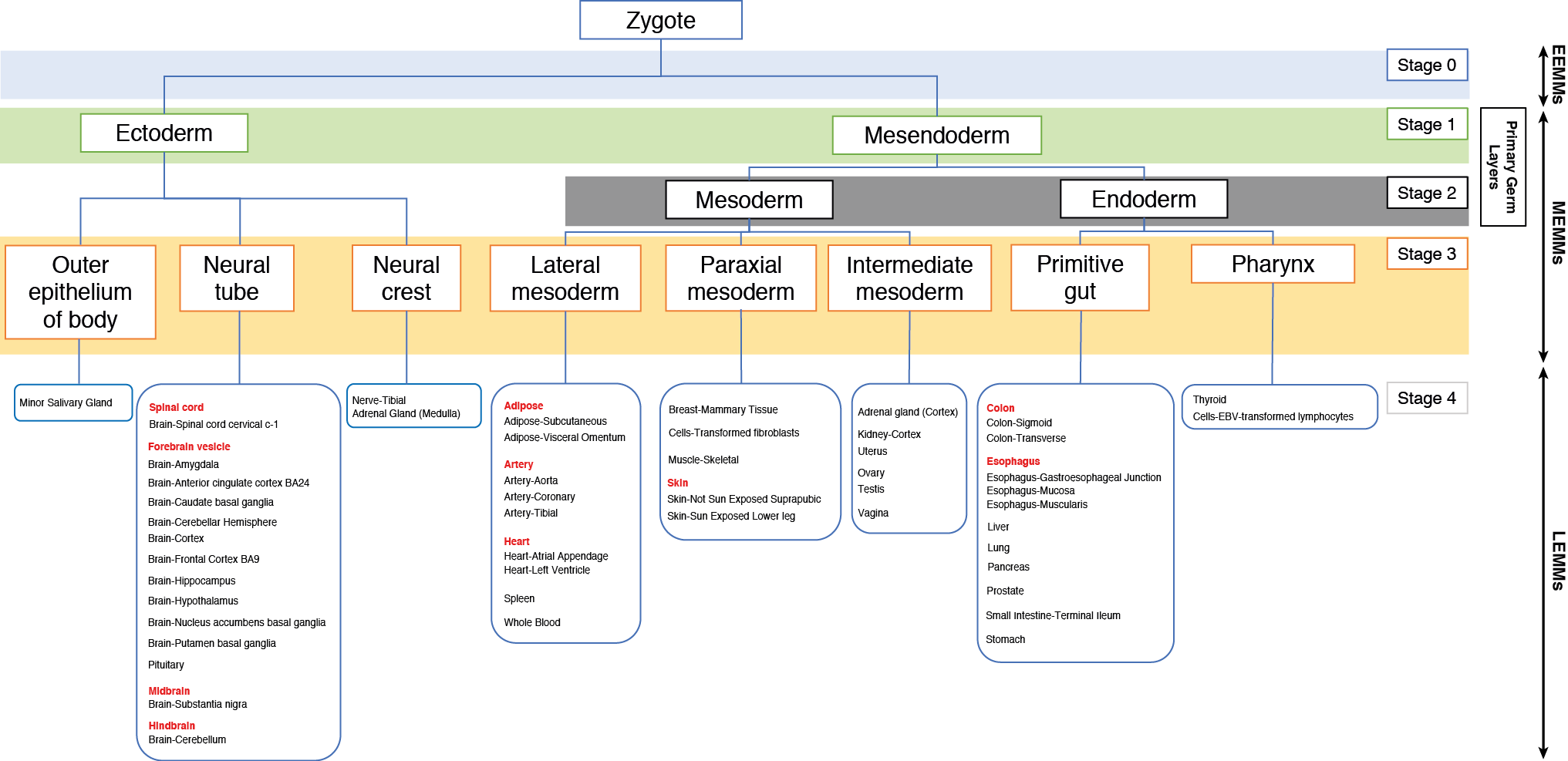


**Fig. S2.** **Lineage tree of human embryogenesis and organogenesis including 49 tissues studied in GTEx**. The *Stage* label groups the tissues based on critical phases of embryogenesis, approximately representing *Stage 0*: from first division of the zygote until late blastulation, *Stage 1*: late blastulation and implantation, *Stage 2*: gastrulation, *Stage 3:* neurulation, and *Stage 4:* organogenesis*.* Black arrows on the right represent the classification of embryonic mosaic mutations into early-embryonic mosaic mutations (EEMMs), mid-embryonic mosaic mutations (MEMMs), and late embryonic mosaic mutations (LEMMs), which has been used throughout this study.

**
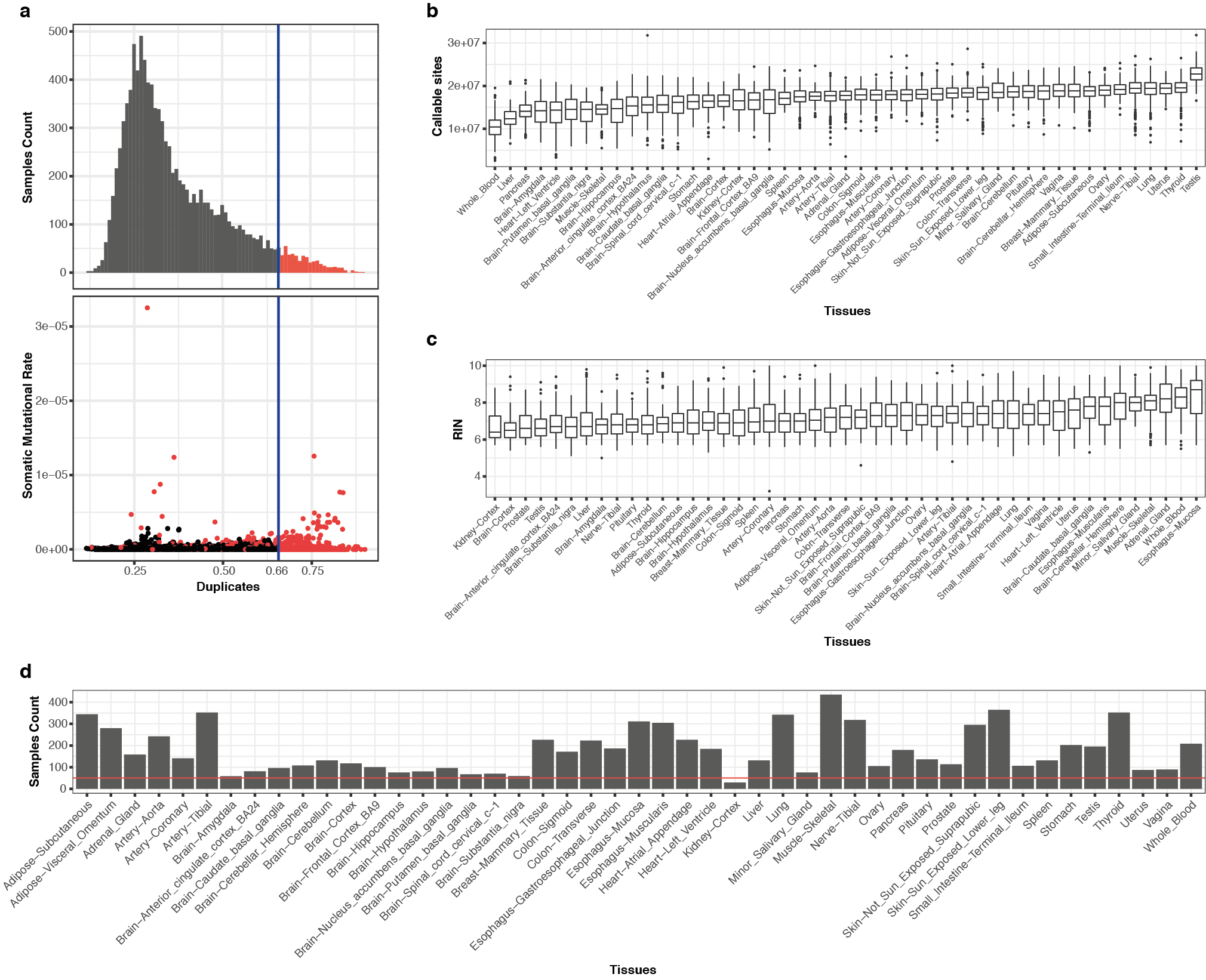
**

**Fig. S3.** **Quality control for RNA-seq data of the GTEx cohort for somatic mutation analysis.** (a) Samples with high PCR duplication rate have been excluded, as duplication rate positively correlates with the number of predicted somatic mutations (confounder), (b) number of callable sites per tissue is depending on the number of expressed genes per tissue, (c) Distribution of RIN values per tissue. (d) Number of sequenced individuals per tissue post QC filtering. Tissues with less than 50 samples (below red line) were removed from the study (only kidney).

**Fig S4.** Early embryonic mosaic mutation rates stratified by gene expression levels in early embryogenesis. Embryogenic expression levels were obtained from Yan et al. 2013 using samples up to the 8-cell embryo state (RPKM values). Numbers below bars show the number of genes included in each group. No significant correlation was found between RPKM values and the mutational rates (R^2^ = 0.009 and p-value = 0.94).


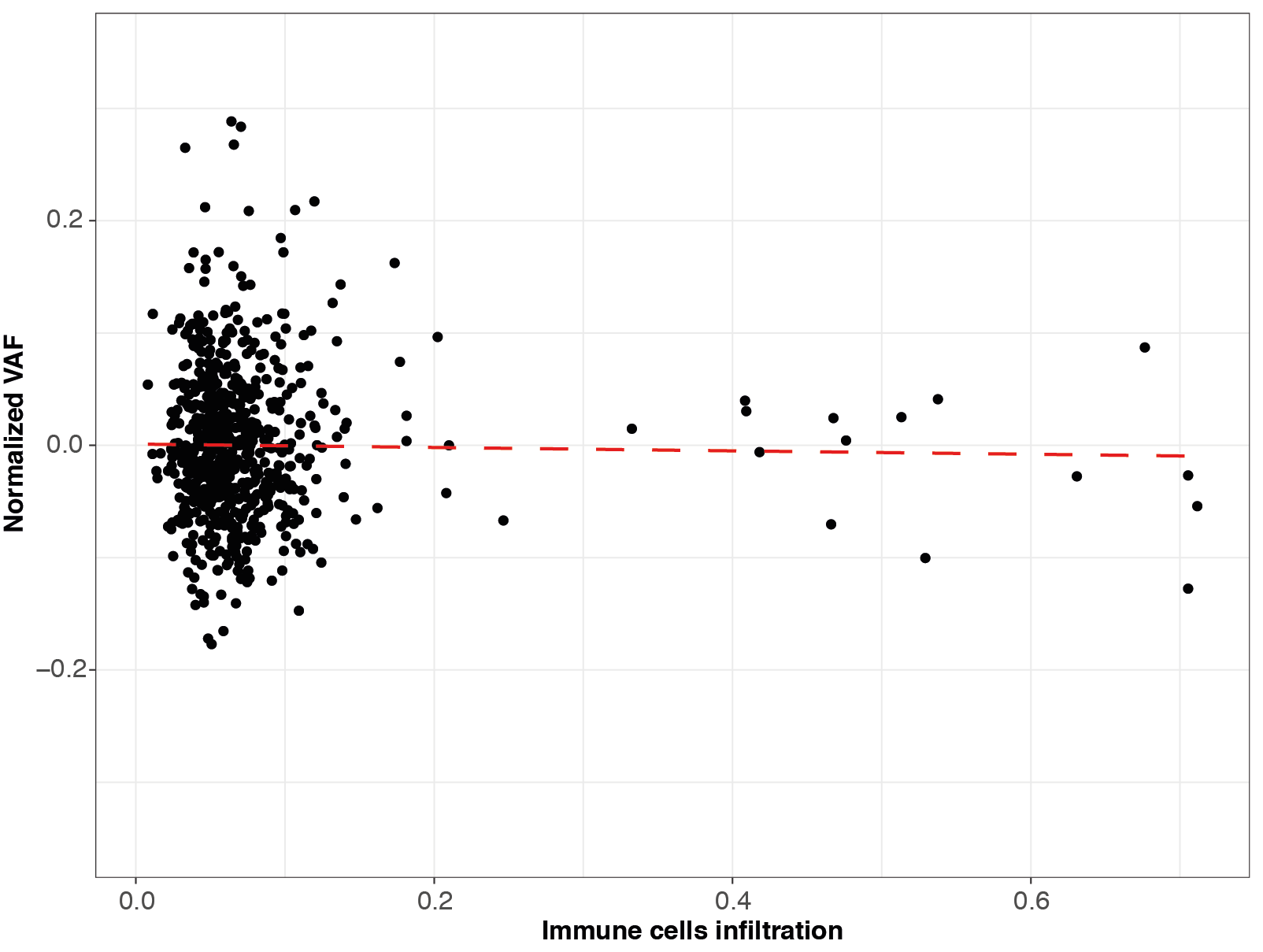


**Fig. S5.** Comparison of variant allele frequencies (normalized VAFs) of embryonic mosaic mutations and the fraction of immune cells in the respective sample harboring the mutation. Pearson’s test showed no significant correlation (R = -0.017, p-value = 0.648).

**
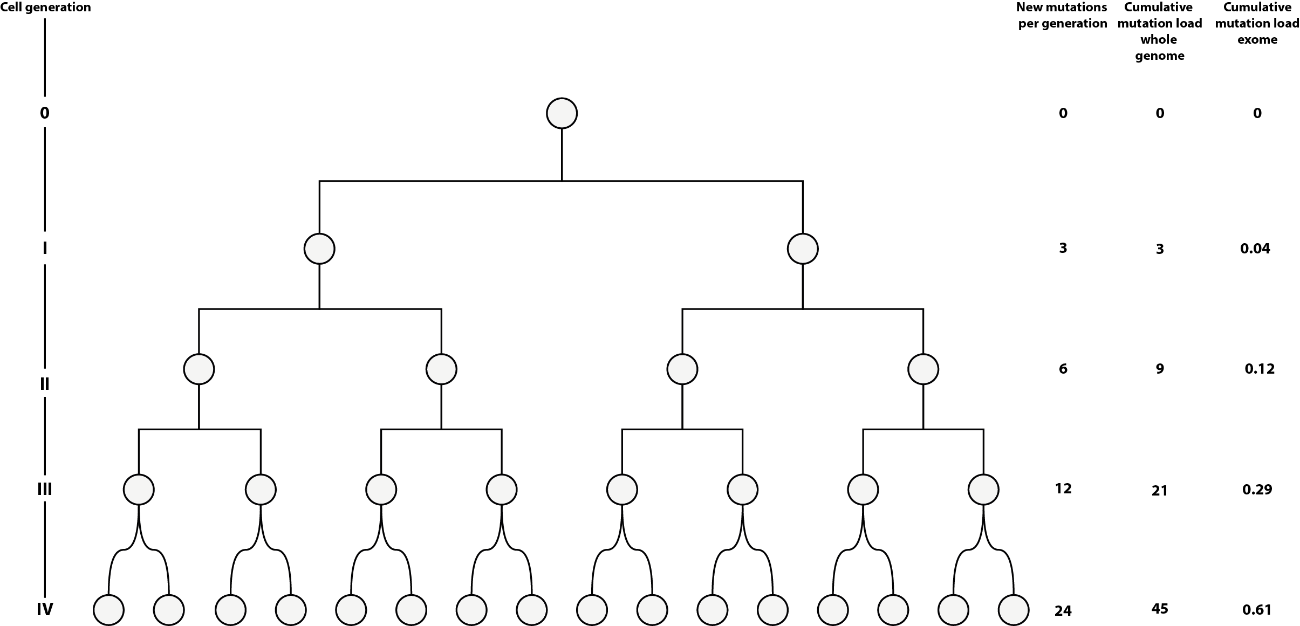
**

**Fig. S6.** Estimation of early mosaic mutation burden in exons (exome) after 1 to 4 divisions of the zygote based on the estimated number of mutations per cell division obtained by whole genome sequencing of blood samples from 241 individuals (Ju 2017). Ju et al. reported that approximately three point mutations per cell per cell-doubling event are acquired during early human embryogenesis. On the right side of the plot we show the expected cumulative mutation load across the whole genome and exome in each cell generation. We found that the expected exome-wide mutation load for the 3^rd^-4^th^ cell generation is similar to our estimated EEMM burden for exomes.

**
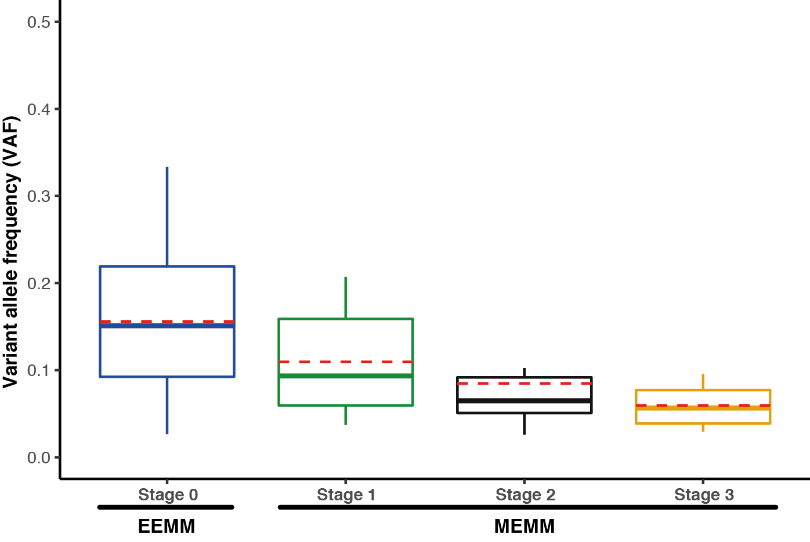
**

**Fig. S7**. **Variant allele frequency (VAF) distribution of mosaic variants mapped to different stages of embryogenesis** (see Fig. S2). Early-embryonic mosaic mutations (EEMM) showed greater VAFs than mid-embryonic mosaic mutations (MEMM). The distinguishable stages of embryogenesis show a significant correlation with VAF when considering stages 0-3 described in Fig. S2 (Spearman correlation (Rho) = -0.39 and p-value of 7.827 x 10^-5^), as well as when considering only MEMMs, i.e. stages 1-3 (p-value = 0.0460 and rho = -0.3301).

**
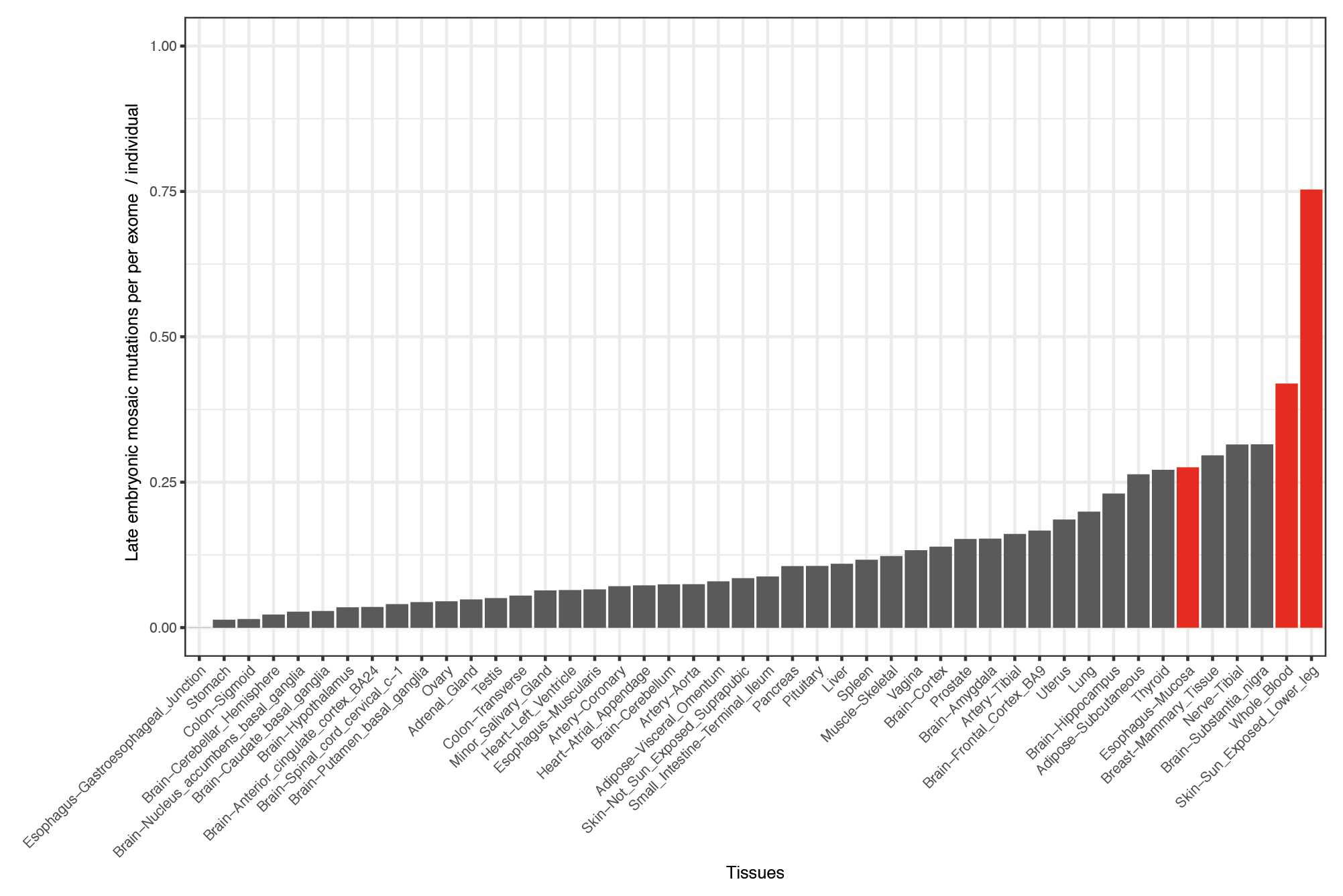
**

**Fig. S8**. **Rate of late embryonic (organ-specific) mosaic mutations observed per tissue and individual in human coding regions (45 Mbps)**. Values were normalised by the number of informative samples per tissue. Red tissues were excluded in the identification of LEMMs, as they have been reported to harbour detectable clonal expansions.

**Fig S9.** Heatmap showing gene expression values (TPM) across different tissues for a set of 100 single-tissue mutations randomly selected across all individuals. Black boxes represent the tissue in which the mutation has been detected (somatic SNVs). Expression levels (TPM) for the other tissues of the individual can range from low (blue) to high (red). White boxes indicate that no expression data was available in the GTEx dataset.


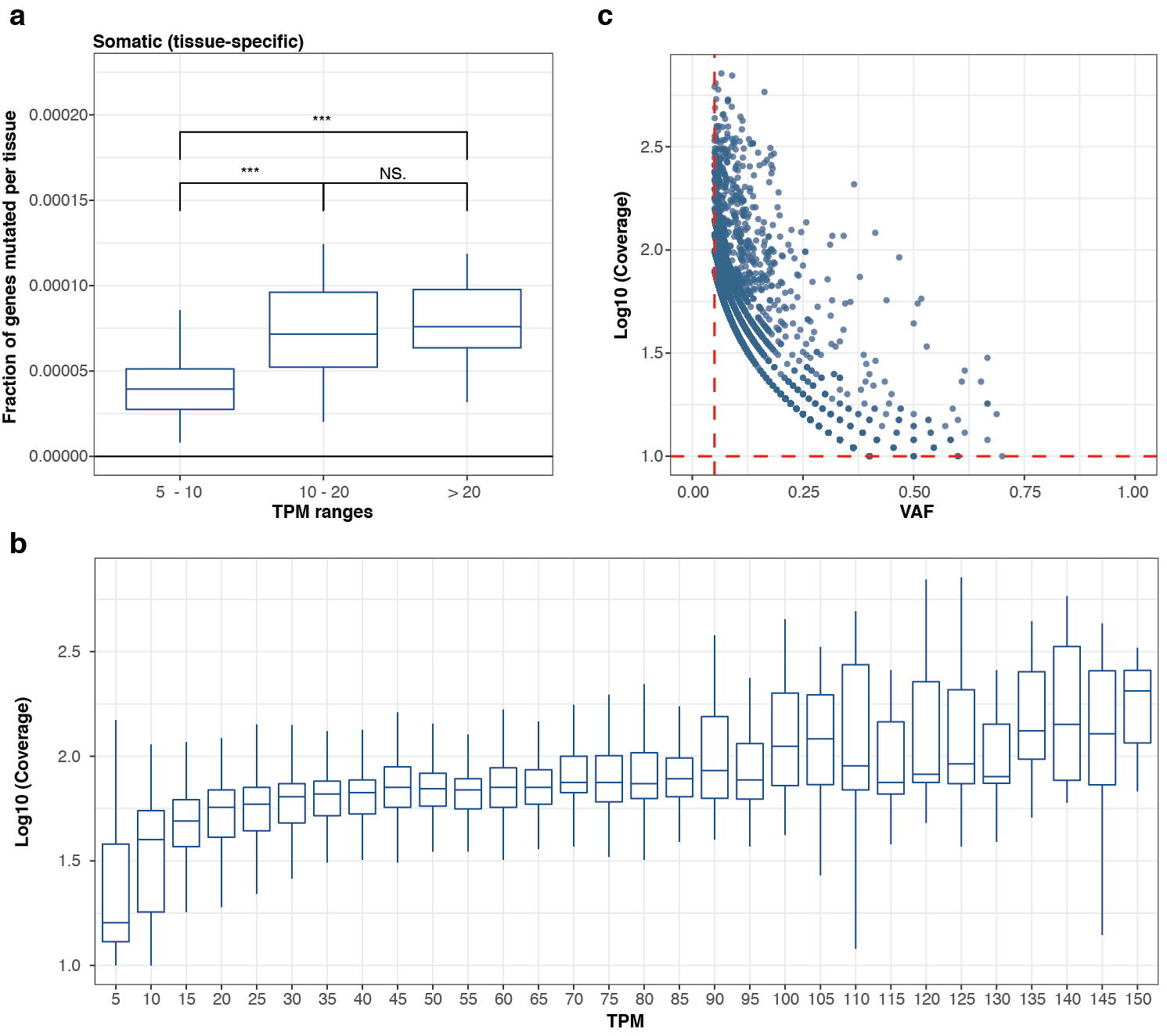


**Fig. S10.** Contribution of gene expression levels to the acquisition of mosaic mutations. a) Fractions of genes harboring tissue-specific somatic mutations stratified by gene expression levels (groups: 5-10 TPM, 10-20 TPM, >20 TPM). Groups were compared using Student’s t-test. b) Depth of coverage (log_10_) correlates with gene expression levels measured in TPM. c) Variant allele frequencies (VAFs) as a function of coverage (log_10_) for tissue-specific somatic mutations. Detectable VAF levels negatively correlate with coverage (Spearman R = -0.81, p-value < 10^-16^).

**
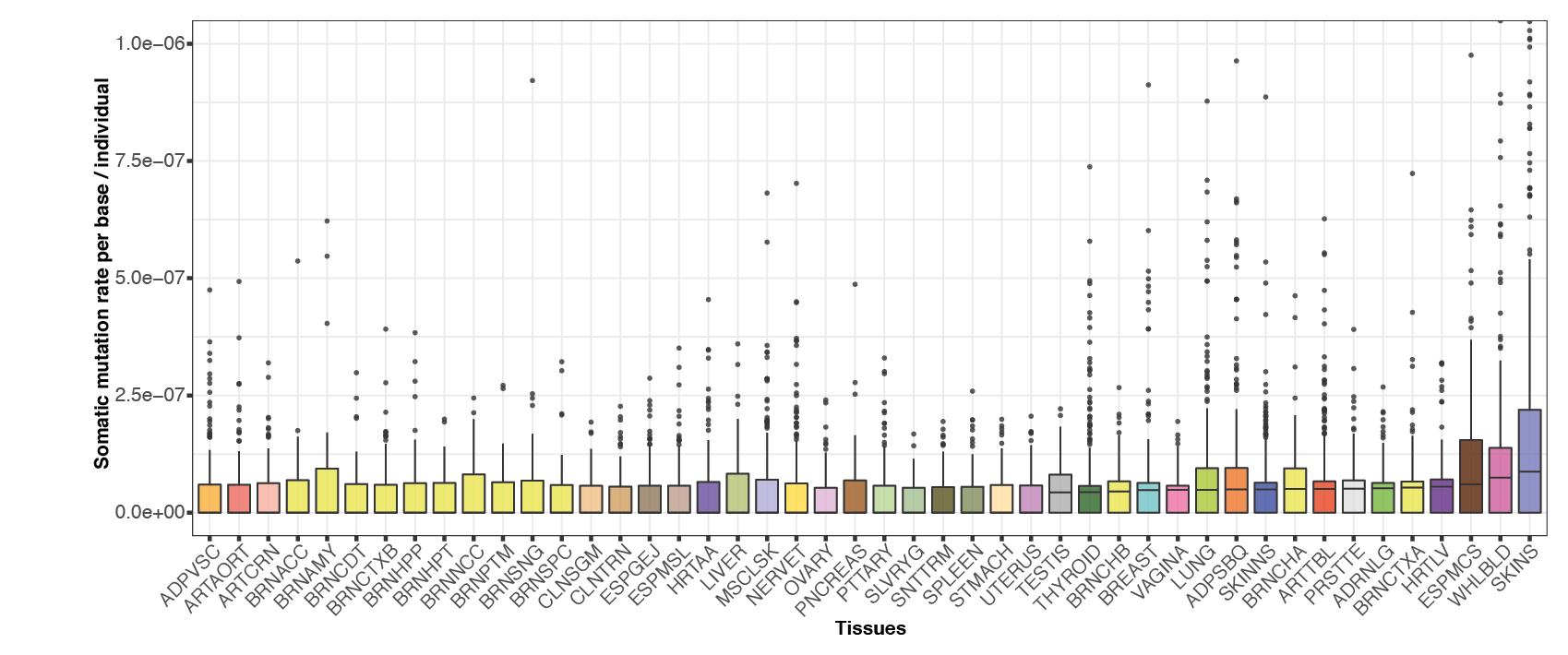
**

**Fig. S11.** **Uncorrected rate of somatic mutations per tissue**. The raw somatic mutations per tissue was found to be confounded by technical biases such as PCR duplication rates, read coverage and RIN. Residuals corrected for technical biases are shown in Fig. 3a.

**
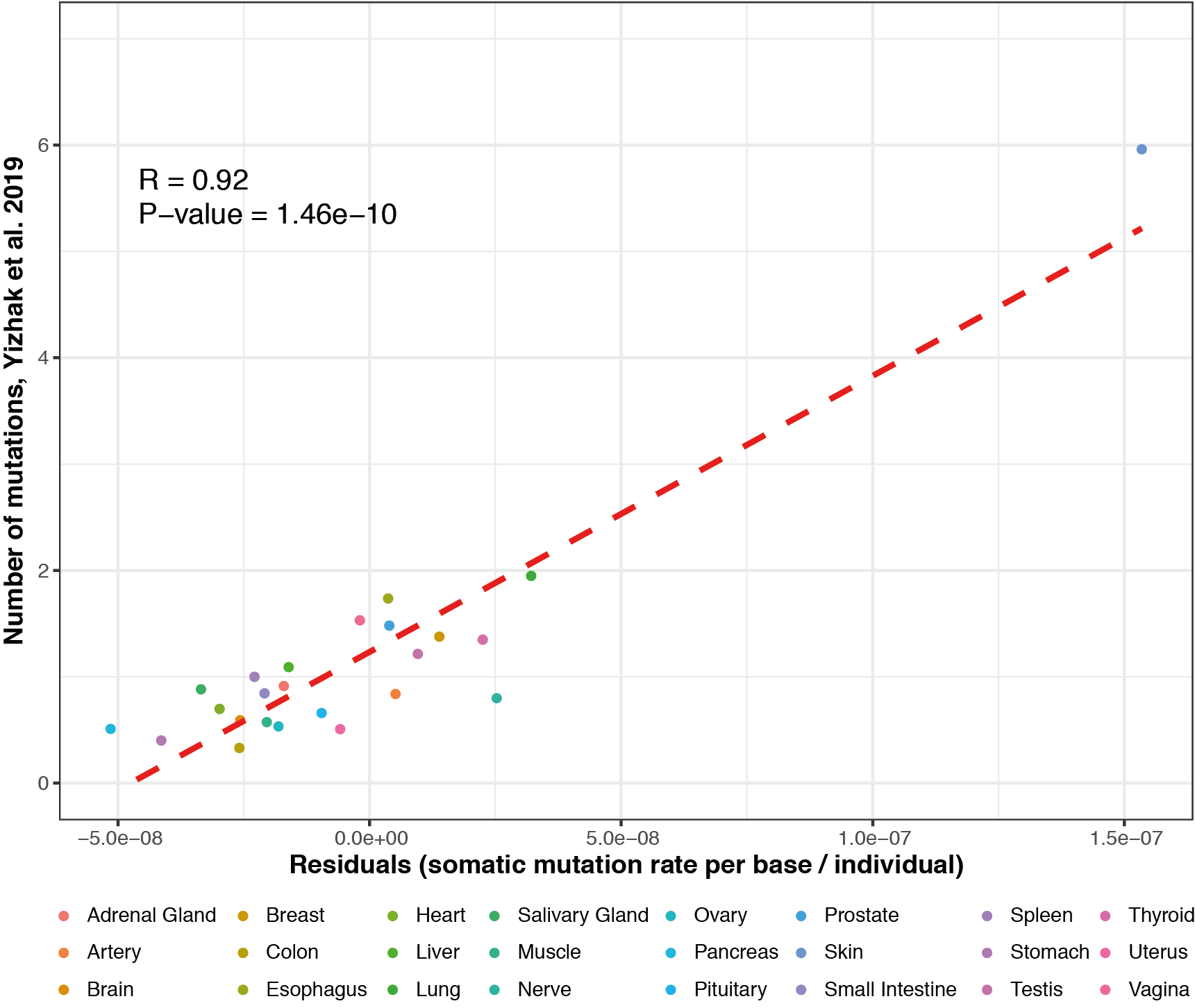
**

**Fig S12.** Comparison of the mean mutation loads per sample for 24 tissues reported in Yizhak et al. 2019 and in our study. Tissues were grouped as in Yizhak et al. 2019. We observed a highly significant positive correlation between mutations described in both studies (Pearson R = 0.92, p-value = 1.46 x 10^-10^).

**
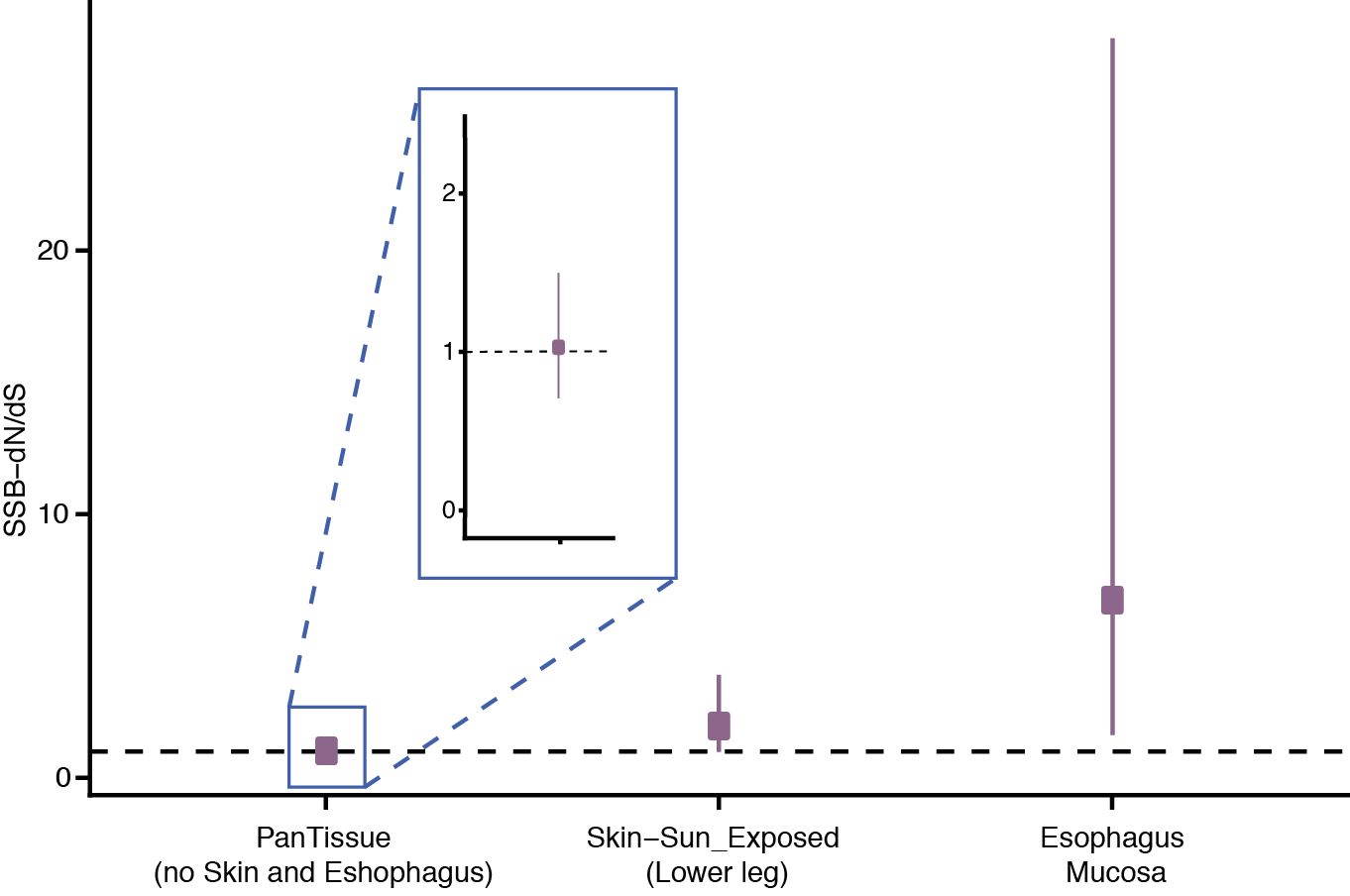
**

**Fig. S13. Signatures of positive selection in cancer genes identified for sun-exposed skin and esophagus-mucosa**. Pan-tissue analysis (without skin and esophagus-mucosa) revealed neutral evolution for the majority of tissues.

**
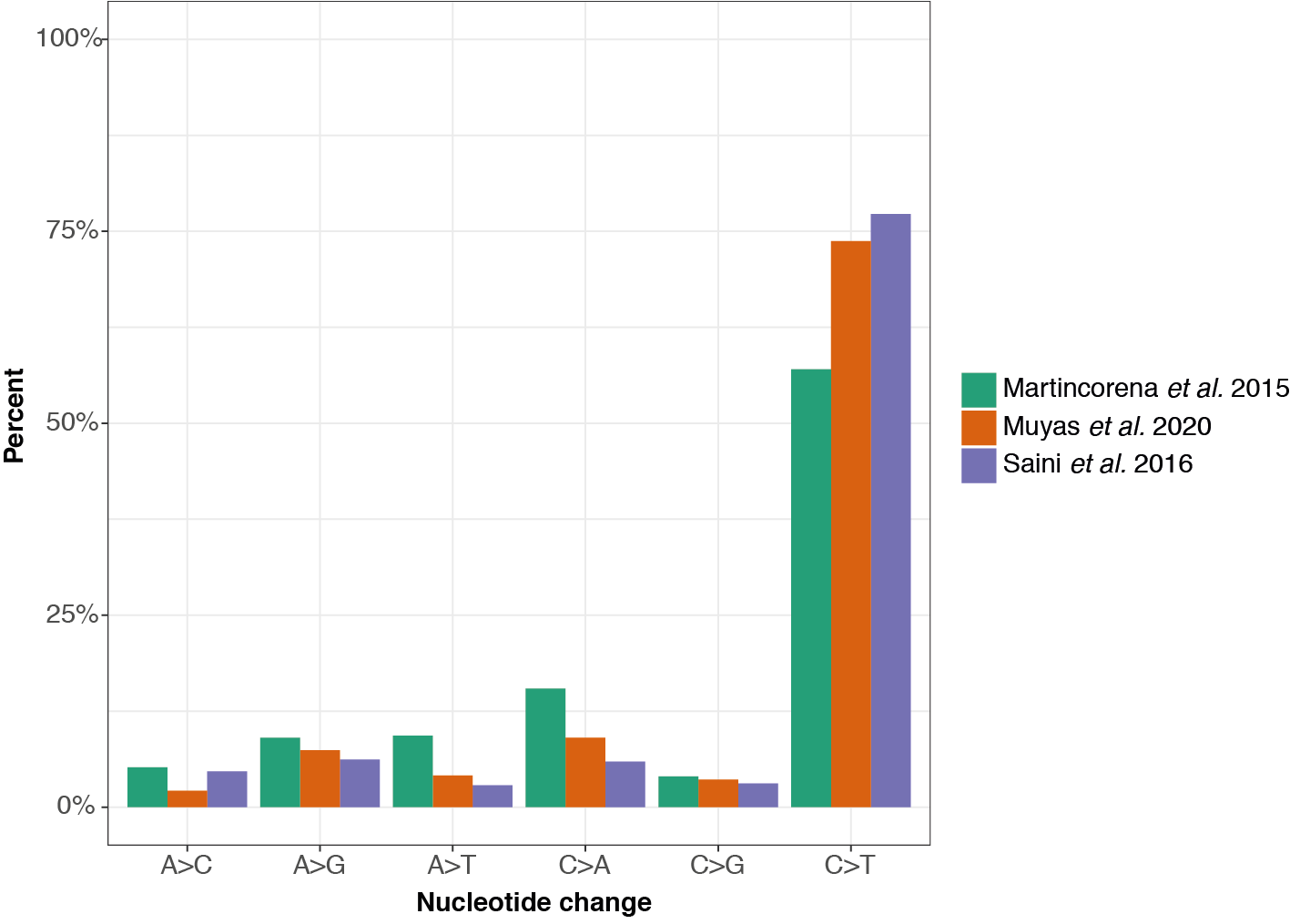
**

**Fig. S14**. Distribution of nucleotide change types for tissue-specific somatic mutations detected in skin biopsies of cancer-free individuals, as reported by Martincorena et al. 2015, Saini et al. 2016 and our study (Muyas et al. 2020). Each study is based on a different cohort of test persons.


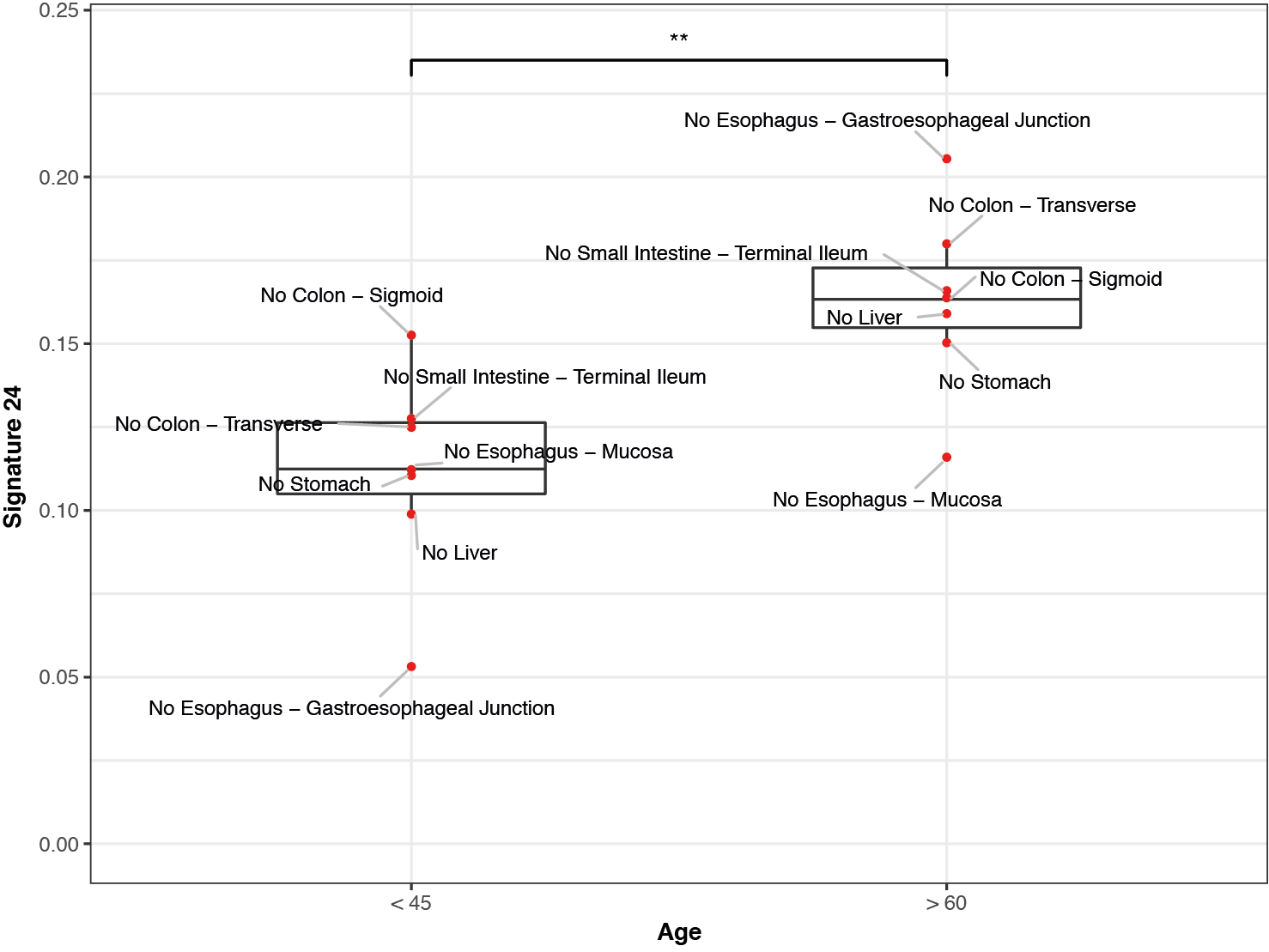


**Fig. S15. Comparison of signature 24 weights (aflatoxin signature) in tissues of the GI tract between younger (< 45 years old) and older (> 60 years old) individuals.** Signature values were recalculated for each group by leaving out one tissue of the GI tract at a time. We found a significant difference between the two groups (two-way Mann-Whitney-Wilcoxon’s test, p-value < 0.01 **).
