# Supplementary material for: The rate and spectrum of mosaic mutations during embryogenesis revealed by RNA sequencing of 49 tissues
Source: Genome Med. 2020 May 27;12:49. doi: 10.1186/s13073-020-00746-1 (PMC7254727; doi:10.1186/s13073-020-00746-1)
Supplement: Supplementary file 5 — Additional file 5. Supplementary methods and extended results. [file 13073_2020_746_MOESM5_ESM.docx]

**Additional file 5**

**Supplementary methods and extended results**

**Benchmarking of the variant call filter *RF-RNAmut* using permutation tests**

In order to test the performance of *RF-RNAmut* in filtering false positive SNV calls we compared it to random forest (RF) models trained on permuted training data. To this end, we permuted the labels (true, false) of the training set, while keeping the feature data unchanged. Using ICGC-CLL data, for which we had a labeled set of true positives from whole-exome sequencing data, we generated 500 permuted RF models on different permutations of the labels. Subsequently, we tested the 500 permuted-RF models and *RF-RNAmut* on an independent test set (subset of ICGC-CLL mutations not used during training).

The precision of the raw SNV calls was 54%. None of the 500 permuted-RF models could improve the precision significantly (Fig. S1, black distribution compared to the red dashed line). In contrast, *RF-RNAmut* increases precision from 54% to 85% (blue dashed line in Additional file 4: Fig. S1).

**Correlation of estimated embryonic mutation rates with embryonic gene expression levels**

Gene expression levels influence transcription-coupled repair efficiency. In order to measure if transcription-coupled repair efficiency influenced our estimates of EMMs we aimed at comparing the estimated mutation rates between stratified sets of low, medium and highly expressed genes. For estimation of EMM rates we had chosen 7630 genes, which were highly and constitutively expressed in most of the analyzed adult tissues. However, using a gene expression atlas for early embryogenesis [44] we found that a substantial fraction of these genes were lowly expressed during the first divisions of the zygote. We could therefore compare the estimated mutational rates in the following three groups of genes stratified by low (0-5 RPKM), medium (5-10 RPKM) and high (>10 RPKM) expression during early embryogenesis (Additional file 4: Fig. S4). We observed no significant correlation between embryonic gene expression levels and EEMM mutational rates (R^2^ = 0.009 and p-value = 0.94), indicating that transcription-coupled repair efficiency at different expression levels had no measurable effect on the estimation of mutational rates. The estimated rate was found to be highly similar in the low and the high gene expression set, supporting our hypothesis that EMMs are mainly driven by mutational signature 1, while signatures related with transcriptional bias or transcription-coupled nucleotide repair (signatures 4, 7, 11, 24 and 25) were absent in EMMs.

**Correlation of immune cell infiltration levels and VAFs of embryonic mosaic mutations**

To interrogate if our estimates of the frequency of EMMs were biased by the fraction of immune cells in a focal tissue or sample (termed ‘immune cell infiltration’), we first computed the fraction of immune cells for each sample using the tool EPIC [63] based on the RNA-seq data. We ran EPIC with TPM values per gene and sample as input using default EPIC parameters. Considering EPIC’s immune cell infiltration measures per sample, we compared the VAF of each EMM with the respective immune cell infiltration level of the sample harboring the mutation.

This comparison could reveal if high levels of immune cells influence the VAFs of the mosaic mutations, representing a contamination with hematopoietic lineage-specific mutations carried in the immune cells. However, we observed no correlation between VAFs and the immune cell fraction of samples (p-value = 0.648, Pearson correlation’s test, new Additional file 4: Fig. S5), evidencing that infiltration of hematopoietic cells did not measurably bias our results.

**Comparison of estimated rates of early-embryonic mosaic mutations with estimates reported in previous studies**

To our knowledge, our estimation of the early-embryonic mosaic mutation (EEMM) rate is the first to be obtained using multiple tissues from the same individual. However, several studies based on single tissues have estimated the mutation rates in human cells. Ju and colleagues (Ju 2017) used data of 241 sequenced blood samples to estimate that humans accumulate approximately 3 SNVs per cell per cell-doubling event across the whole genome during early embryonic development. Considering this estimate, we calculated that the cumulative number of EEMMs after the first 3 to 4 cell divisions of the zygote sums up to around 21 to 45 mutations in the whole genome and 0.29 to 0.61 mutations per exome (45 Mbps) per embryo (Additional file 4: Fig. S6). This estimate, although based on a single tissue, was surprisingly similar to the one we have estimated for EEMMs in the analysis of our cohort. We therefore claim that the obtained estimates are robust at least for very early embryonic mutations, which can be reliably detected across multiple tissues.

**Completeness of mosaic mutation calls across tissues defines the accuracy of distinguishing single-tissue mutations from early embryonic mutations affecting multiple tissues**

The accuracy of determining if a mutation affects exactly one tissue is limited by the completeness of the GTEx tissue matrix and low expression of genes in a subset of studied tissues. However, for estimating the frequency of early and mid-embryonic mosaic mutations it is only important to ascertain if a mutation occurred before neurulation or not (see Additional file 4: Fig. S2). To do this reliably we do not need high read coverage for a focal genomic position in each of the 49 investigated tissues, as long as sufficient coverage is available for at least a few tissues of each primary germ layer. Hence, we examined the gene expression levels across tissues for a set of 100 randomly selected single-tissue mutations (Additional file 4: Fig S9). For most variants we observed high gene expression levels in more than 50% of the tissues, but always in more than 30%. We conclude that for most of the mutations a sufficient fraction of tissues showed enough high expression to determine that they did not occur before neurulation. Nonetheless, it is possible that some mutations labeled as late embryonic (affecting only one organ) have occurred earlier than estimated and affect other tissues for which we have missing data. However, if a mutation was found in at least two tissues originating from different germ layers, it very likely occurred before neurulation. This means that the estimated EMM rate is a lower boundary, i.e. most likely not overestimated.

**Correlation analysis of somatic mutation rate and gene expression levels**

Previous studies have indicated that expression levels influence the frequency of mutations, which could be explained by the effectiveness of transcription coupled repair or changes in chromatin structure between active and inactive regions of the genome. We therefore tested, if the level of gene expression, measured in transcripts per million (TPM), correlates with the fraction of mutated genes. To this end, we stratified genes by expression levels using the TPM ranges [5 – 10), [10 – 20) and > 20, and compared the fraction of genes harboring tissue-specific somatic mutations in each group. We observed a lower fraction of somatic mutation carrying genes for the lowest TPM range (Additional file 4: Fig. S10a).

However, we cannot discard the likely hypothesis that reduced fraction of mutated genes at low TPM is due to a lower power of detection of somatic SNVs in lowly expressed genes. As expected, lowly expressed genes showed lower coverages with the most dramatic drop of coverage below 10 TPM (Fig. S8b). Moreover, when using a cutoff of 4 alternative reads for variant calling (see Methods), the detection limit for somatic mutations at very low TPM is >25% VAF (Additional file 4: Fig. S10c), compared to the 5% VAF detection limit we achieve for TPM > 20.

Closer inspection of Additional file 4: Fig. S10b reveals that 5 TPM corresponds to a median read coverage of 15, while 10 TPM and 20 TPM correspond to median coverages of 40 and 55, respectively. This massive drop of coverage below 10 TPM likely explained the reduced detection power and the reduced frequency of mutated genes in the low TPM range. Indeed, we found now difference in the fraction of mutated genes between the expression ranges 10-20 TPM and >20 TPM (Additional file 4: Fig. S10c). We therefore conclude that no significant difference can be found for medium to highly expressed genes, while the strongly reduced detection limit for lowly expressed genes is strongly confounding the analysis of other effects.

**References**

63. Racle J, de Jonge K, Baumgaertner P, Speiser DE, Gfeller D. Simultaneous enumeration of cancer and immune cell types from bulk tumor gene expression data. Elife. 2017;6:e26476.
